# Evaluation of behavior in veal calves fed milk containing different levels of hempseed cake (*Cannabis sativa L*.)

**DOI:** 10.3389/fvets.2023.1295949

**Published:** 2023-12-20

**Authors:** Sheyla Arango, Nadia Guzzo, V. Trabacchin, Emiliano Raffrenato, Cristina Sartori, Lucia Bailoni

**Affiliations:** ^1^Department of Comparative Biomedicine and Food Science (BCA), University of Padova, Legnaro, Italy; ^2^Department of Agronomy, Food, Natural resources, Animals and Environment, University of Padova, Legnaro, Italy

**Keywords:** animal behavior, calves, hempseed cake, hemp, nutrition

## Abstract

The present study aimed to evaluate the effect on behavioral patterns of veal calves fed with increasing levels of hempseed cake (HSC) diluted in the milk replacer. In total, 48 Belgian Blue veal calves (12 females and 36 males), with a body weight (BW) of 62.0 kg and age of 42.6 days, were offered the same type and quantity of solid and liquid feed two times a day but randomly assigned to one of the three different HSC inclusion levels: 0% (CTR), 3% (T3), and 6% (T6). The study lasted for 6 months until slaughter. During this time, their behavior was recorded using video cameras provided with a surveillance system. The results indicated that HSC had negligible effect on calves' behavior and that calves, in general, spend most of their time resting and ruminating as they normally do with conventional diets. Hempseed cake inclusion (T3 and T6) increased (*P* < 0.05) the appetite for solid food and licking behavior during the late afternoon. T3 female calves increased (*P* < 0.05) their movement in the late afternoon. Male calves decreased (*P* < 0.05) their positive interaction, movement, and cross-sucking in the late afternoon as the inclusion of HSC increased. The inclusion of HSC into veal calves' diet did not negatively affect the animal's behavior; therefore, it can be suggested as a novel ingredient.

## 1 Introduction

*Cannabis sativa* L., commonly known as hemp, has been primarily grown for its fiber (1), but over the last 10 years, it has also been attracting some interest in the animal feeding sector. Hemp by-products, such as oil, seeds, and cake, have large amounts of polyunsaturated essential fatty acids, particularly linoleic and alpha-linolenic, which make them suitable ingredients for the formulation of animal feeds. Fish and farm animals such as laying hens, broiler chickens, pigs, sheep, and other ruminants are continuously being tested in order to establish the range of inclusion levels that ensures animal health and also leads to optimal performances (2).

An interesting farm production system is that of white veal meat. Italy is the fifth major producer of this type of meat in the European Union, owning a population of 378,459 calves in 2022 (3). White veal calves must be younger than 8 months old at slaughter and are raised to obtain pale meat based on a low-iron milk replacer with the addition of concentrate (4). For this purpose, the use of hempseed cake (HSC) in their diets could represent a good protein source.

Although industrial hemp is characterized by having trace amounts (<0.30%) of tetrahydrocannabinol (THC) (5), which has psychotropic effects, it cannot cause intoxication. Cannabidiol (CBD) is the second bioactive compound in hemp, and it is known for having remarkable applications without psychoactive effects (6). In fact, CBD extract from *C. sativa* has been tested in dogs and seems to reduce aggressive behavior (7). Moreover, chronic treatment with non-psychotropic *C. sativa* caused no alterations in body weight, movement, or anxiety in mice, while increasing pro-social behavior (8). Considering that research on the behavioral effects of hemp is fairly recent, no studies have focused on farm animals to date. In addition, since consumers, at present, are increasingly concerned about animal welfare issues (9), it is always necessary to test new feed sources in order to understand any possible behavioral changes before the feed becomes part of the calves' diet. This study aimed to evaluate the effect on behavior patterns of veal calves fed with increasing levels of hempseed cake diluted in the milk replacer.

## 2 Materials and methods

All procedures were performed according to the Italian legislation on animal care and approved by the Ethical Committee for the care and use of experimental animals at the University of Padova, which operates within the European Directive 86/609/CEE regarding the protection of animals used for experimental and other scientific purposes (approval number 74/2022).

### 2.1 Housing system and experimental design

This trial was carried out at a commercial farm (BE farm, owned by Barban Elia, Castelfranco, TV, Italy) and aimed at evaluating the differences in behavioral patterns when Belgian blue calves were fed with increasing levels of hempseed cake in the milk replacer. Data for this study were obtained from 6 months of the fattening period of 48 Belgian Blue veal calves (12 females and 36 males). The number of male and female calves was chosen after sample size calculation using a Student's *T*-test with a statistical power of 0.75 and a statistical significance of α = 0.05. The animals were randomly distributed in 12 pens (four calves per pen); after the random assignment, some changes were made to balance the pens for body weight (BW). There were nine pens (three per treatment) for male and three for female (one per treatment) calves. Each pen had a dimension of 3 × 2.5 m. The average age on arrival was 42.6 ± 9.5 days, and the average BW was 62.0 ± 4.5 kg. Animals were reared for 6 months until slaughter.

**Figure 1:**
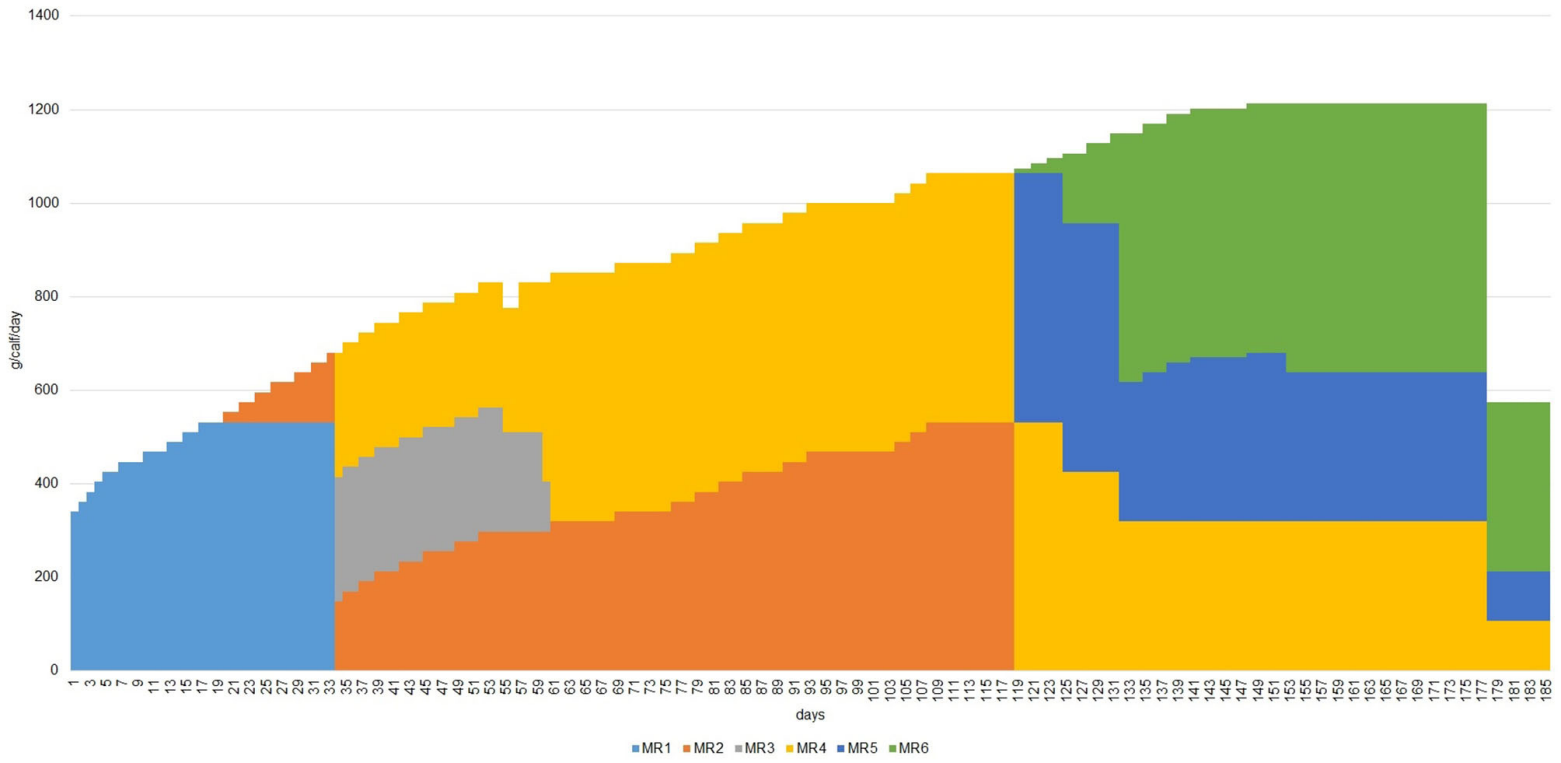
Milk replacers (MR1, Elvor Demarrage 50; MR2, Zoogamma M-21; MR3, Elvor Ingrasso 50; MR4, Unico Super I; MR5, Top 60; MR6, Elvor Finition 50 N) intake (g/calf/day) during the experiment.

The diet consisted of solid and liquid feed (Table 1), and all animals received the same amount of each feed. The solid feed used was Fibra Flakes 314 PN concentrate (Veneta Fiocchi, Riese Pio X, TV, Italy). The liquid feed followed the typical program used for white veal calves by the farm, where the trial was conducted and consisted of six types of milk replacers (Sofivo, Maen Roch, France) that were offered throughout the experiment (Figure 1) until weaning, from 340 g on day 1 to 1,213 g before slaughter in the sixth month of the trial. The three experimental diets (CTR, T3, and T6) differed in the percentage of hempseed cake (as feed) diluted in the milk replacer and were based on the EFSA daily recommendation for hemp by-products in ruminants (2). The CTR group had no inclusion of HSC. T3 had an inclusion of 3% (as feed) of HSC, corresponding to 7.5 g on day 1 and 750 g before slaughter. T6 had 6% (as feed) of HSC inclusion, corresponding to 16 g on day 1 and 1,500 g before slaughter. The chemical composition (% of DM) of the HSC was 26.17 of CP, 11.89 of EE, 11.89 of CF, 0.014 of THC, and 27.31 of iron (mg/kg DM). Liquid feed was prepared freshly before each meal by mixing the milk replacer with water and then with the HSC. It was provided two times a day (at 6.00 a.m. and 5.00 p.m.) on individual teat buckets placed in each pen. Solid feed was always provided after the liquid feed and also twice a day in increasing amounts from 75 g on day 1 to 2,400 g at the end of the trial. Fresh water was offered *ad libitum* using a drinking cup placed in the corner of each pen.

**Table 1:** Chemical composition of the concentrate (CON) and the six milk replacers (MR) given to all the calves.

**Item**	**CON**	**MR1**	**MR2**	**MR3**	**MR4**	**MR5**	**MR6**
**Chemical composition, % of DM**							
Dry matter	88.80	96.5	95.84	95.69	96.16	96.01	96.54
Crude protein	14.49	19.88	21.40	21.72	17.20	23.46	21.18
Ether extract	4.07	16.50	22.99	23.27	16.74	20.31	21.93
Non-structural Carbohydrates	77.98	56.67	48.31	47.81	58.48	49.14	50.45
Ash	3.46	6.95	7.30	7.21	7.59	7.09	6.44
Iron (mg/kg)	55.83	50.4	7.21	6.39	28.36	26.04	6.12

CON, concentrate; MR, milk replacer; MR1, Elvor Demarrage 50; MR2, Zoogamma M-21; MR3, Elvor Ingrasso 50; MR4, Unico Super I; MR5, Top 60; MR6, Elvor Finition 50 N.

### 2.2 Behavioral observations

The behavior of the calves was recorded using a video surveillance system [H.264 Standalone Digital Video Recorder (DVR); Atlantis, Atlantis-land, MI, Italy]. A total of 12 cameras were fixed on the ceiling in front of each pen in order to cover the whole area. The cameras recorded 24 h of 3 days per week from the beginning of the trial, but only 1 day per week (the same day each week) was chosen for this study. The videos were analyzed by two different viewers using the *Playback Software* program (Atlantis, Atlantis-land, MI, Italy), which allowed them to observe the behavior of the animals during the entire target day. In total, 432 h were analyzed (216 h per viewer). They were taken for 18 days, starting approximately a month after the administration of the experimental diets. The ethogram was built by looking at three sample hours of the whole day and considering the behaviors found in the veal calves during the trial. Behaviors were classified according to the literature (10), and a total of 24 behaviors were identified (Table 2). Each behavior was ascribed to one of the following categories: ingestion (three behaviors), resting (three), ruminating (three), movement (four), environmental interaction (three), positive interaction (four), negative interaction (three), and self-grooming (one). A letter (A, B, C, or D) was assigned to each calf within a pen for unique identification. After a previous validation of the scan sampling interval, every 5 min, the behavior of each calf was registered in an Excel spreadsheet. To appreciate the variability of the behaviors over the 24 h of a day, the day was divided into six time intervals (timings) of 4 h each: night (NI, 12.00 midnight−4.00 a.m.), early morning (EM, 4.00 a.m.−8.00 a.m.), late morning (LM, 8.00 a.m.−12 noon), early afternoon (EA, 12.00 noon−4.00 p.m.), late afternoon (LA, 4.00 p.m.−8.00 p.m.), and evening (EV, 8.00 p.m.−12.00 midnight). Each behavior was considered as a trait and was expressed as the percentage of recordings of such behavior collected from an individual within a target timing. Specifically, the number of occurrences of a specific behavior recorded for each calf within a specific timing of a target day was divided by the total number of recording occurrences within a timing and then multiplied by 100. In such a way, each individual behavior was expressed as a percentage of recordings within the respective timing. The final dataset included 5,184 records of each individual single behavior within a day of observation and specific timing.

**Table 2:** Ethogram of calves' behavior within categories and main description of each behavior.

**Behavioral categories**	**Behavior**	**Description**
Ingestion	Eating solid	Ingestion of the solid feed from the feeder
	Eating liquid	Ingestion of the milk replacer
	Drinking	Drinking from the drinking cup in the corner of the pen
Resting	Sternal recumbency	Resting or sleeping with the legs curled under the body and the head up
	Lateral recumbency	Resting or sleeping with the legs and head outstretched
	Rest standing	Standing inactive in a relaxed posture; head lowered, eyes partially or totally closed
Ruminating	Standing ruminating	Chewing motions of teeth while standing on all four legs
	Lateral ruminating	Ruminating with the legs and head outstretched
	Sternal ruminating	Ruminating with the legs curled under the body and the head up
Movement	General agitation	Walking beside the feeder from one side to another with or without a reason, mostly close to the feeding times
	Standing/lying	Passing from standing to lying or vice versa
	Moving	Displacement slowly from one location to another inside the pen
	Running	Rapid movement with constant changes of direction inside the pen
Environmental interaction	Olfactory investigation	Sniffing various parts of another individual's head or body, which typically begins after a nose-to-nose approach
	Object playing	Playing with an object inside the pen
	Licking	Licking the wall, the empty feeder, or some object inside the pen
Positive interaction	Mutual grooming	Grooming and licking another individual using gentle gestures
	Playing	Playing with another calf while making physical contact with their body parts, eventually pushing each other without force
	Pen interaction	Staying near the adjacent pen and exploring by licking the calves there
	Sexual behavior	Mounting; jumping to lift both forelegs onto the rump of another calf
Negative interaction	Cross-sucking	Sucking or licking the perianal zone of another calf
	Stereotypies	Repetitive or unnatural movements with a relative regularity without any apparent function (e.g., tongue playing/rolling and bar biting)
	Head butting	Two calves butting each other with their foreheads and sticking together for some seconds
Self-grooming		A calf licking any part of itself
Non-visible		Not visible from the camera or hidden behind other calves

### 2.3 Statistical analysis

The trial focused on the expression of a specific behavior for each diet (CTR, T3, and T6). Each calf represented an experimental unit, and all behaviors were expressed as fractions of an hour. An effect size correlation of r = 0.081 was calculated by comparing control vs. treatment on some behaviors relevant to calf wellbeing, such as cross-sucking, using Cohen's *d* statistics. A separate analysis for each behavior of an individual within each target timing of 4 h (6 timings × 4 h = 24 h) was performed as dependent variables of a mixed model [mixed procedure; SAS, SAS/STAT User's Guide (SAS Institute: Cary, NC, USA, 2013)] in a linear model analysis as written below:


$$\begin{array}{l} {\text{Y}}_{\text{ijklm}}\text{ = μ + }{\text{day}}_{\text{i}}\text{ + }{\text{D}}_{\text{j}}\text{ + }{\text{T}}_{\text{k}}\text{ + }{\text{S}}_{\text{l}}\text{ + }(\text{DxT}{)}_{\text{jk}}\text{ + }(\text{DxS}{)}_{\text{jl}}\text{ + }(\text{TxS}{)}_{\text{kl}}\\ \text{ + }(\text{DxTxS}{)}_{\text{jkl}}\text{ + }{\text{ID}}_{\text{m}}\text{ + }{\text{e}}_{\text{ijklmn}}, \end{array}$$

where Y_ijklm_ is the target individual behavior as a dependent variable and μ is the overall mean. The fixed effects were the day of observation (18 levels, corresponding to each target day), the diet (D: CTR, T3, and T6), the timing (T: EM, LM, EA, LA, E, and N), and sex (S: M or F). The interactions among D, T, and S were also considered. Random effects included the identity of each individual calf (ID) and the error (e). The effect of the two viewers was not included as a separate effect in the final model because it was already considered within the effect of the day, as each day was entirely observed by a single viewer. All the variables and their residuals were tested for normality using a Shapiro–Wilk test. As *post-hoc* analysis of the mixed model, the least square means [LS means option; MIXED procedure, SAS, SAS/STAT User's Guide (SAS Institute: Cary, NC, USA, 2013)] were calculated for each effect included. Additionally, the comparisons between each pair of levels for the LS means of each fixed effect were made using the Student's *t*-test analysis. A Bonferroni correction was done to make it as conservative as possible SAS, SAS/STAT User's Guide (SAS Institute: Cary, NC, USA, 2013). A *P*-value of < 0.05 was used to indicate statistical significance.

## 3 Results

None of the calves developed diseases severe enough to be a reason for exclusion from the study. The descriptive values of the duration of the calves' behaviors are reported in Table 3. During the experiment, some behaviors were identified as belonging to some specific timings. In general, resting and ruminating were the two main behaviors performed by the calves throughout the day. The ingestion behavior was observed mostly during the day. The calves spent 8.4% of their time eating solid food in the late morning and 9.9% in the late afternoon after the administration of the two meals (6.00 a.m. and 5.00 p.m.). As regards the resting behavior, both sternal and lateral recumbency appeared predominantly during the night when the calves just lay down inside the pen. Sternal recumbency was observed in the late morning (38.6%) and early afternoon (40.5%), especially in the evening (53.1%) and during the night (45%). On the other hand, standing resting was barely observed in the evening (5.9%) and night (2.1%), as the calves preferred to rest in the recumbent position. The most common ruminating behavior was sternal ruminating, occurring mainly during the night (22.9%), where the animals remained in sternal recumbency. The environmental interaction of the calves was mostly represented by olfactory investigation and licking. Olfactory investigation toward the other pen mates was observed mainly during the late morning (6.4%) and late afternoon (9.7%). Generally, this behavior occurred at the end of the meal as did licking. The negative interaction behavior in which calves spent the most time was cross-sucking, usually performed in the early morning (1.3%), before the first meal, and in the late afternoon (1.8%) after the evening meal. The other negative behaviors were not strictly linked to a specific time slot but were displayed throughout the day. A few stereotypies were also observed in all the time slots considered.

**Table 3:** Least square means a percentage of recordings of calves' behavior along the day, calculated as the ratio between the number of occurrences of a target behavior and the total recording occurrences in a day and then multiplied by 100.

**Behavior**	**Timing** ^**a**^					
	**EM**	**LM**	**EA**	**LA**	**EV**	**NI**
**Ingestion**						
Eating solid	6.13 ± 5.43	8.36 ± 7.38	1.64 ± 3.42	9.93 ± 5.14	0.93 ± 2.03	0.47 ± 1.48
Eating liquid	2.04 ± 1.35	0.00 ± 0.00	0.00 ± 0.00	2.02 ± 1.35	0.00 ± 0.00	0.00 ± 0.00
Drinking	0.15 ± 0.65	0.29 ± 1.00	0.16 ± 0.68	0.27 ± 0.91	0.14 ± 0.69	0.06 ± 0.37
**Resting**						
Sternal recumbency	28.87 ± 14.42	38.58 ± 17.14	40.45 ± 18.79	17.47 ± 11.37	53.14 ± 21.63	44.85± 21.68
Lateral recumbency	6.60 ± 9.97	7.29 ± 11.20	12.56 ± 14.37	2.31 ± 5.17	12.19 ± 16.41	14.97 ± 17.32
Rest standing	14.48 ± 9.61	11.07 ± 9.11	5.38 ± 5.33	21.37 ± 12.25	5.85 ± 6.70	2.11 ± 2.79
**Ruminating**						
Standing ruminating	0.16 ± 0.75	0.10 ± 0.68	0.05 ± 0.35	0.13 ± 0.73	0.03 ± 0.32	0.05 ± 0.35
Lateral ruminating	2.59 ± 4.73	1.14 ± 3.54	3.14 ± 5.33	0.56 ± 2.16	1.64 ± 3.88	5.68 ± 8.85
Sternal ruminating	13.00 ± 9.74	7.31 ± 7.41	17.24 ± 11.95	6.09 ± 6.08	9.52 ± 9.18	22.94 ± 15.52
**Movement**						
General agitation	0.18 ± 0.59	0.04 ± 0.33	0.01 ± 0.14	0.40 ± 0.87	0.03 ± 0.26	0.00 ± 0.00
Standing/lying	0.20 ± 0.67	0.22 ± 0.67	0.20 ± 0.63	0.17 ± 0.61	0.29 ± 0.76	0.19 ± 0.65
Moving	0.69 ± 1.39	0.80 ± 1.41	0.42 ± 1.05	1.42 ± 2.05	0.40 ± 0.96	0.12 ± 0.53
Running	0.02 ± 0.21	0.08 ± 0.45	0.03 ± 0.28	0.28 ± 0.81	0.05 ± 0.35	0.00 ± 0.00
**Environmental interaction**						
Olfactory investigation	5.86 ± 5.22	6.40 ± 5.64	4.44 ± 4.74	9.68 ± 8.05	3.51 ± 4.58	1.50 ± 2.17
Object playing	0.17 ± 0.90	0.08 ± 0.5	0.05 ± 0.35	0.16 ± 0.72	0.01 ± 0.17	0.00 ± 0.00
Licking	7.61 ± 5.72	6.42 ± 5.59	4.55 ± 5.09	11.69 ± 7.52	2.54 ± 3.66	0.72 ± 1.69
**Positive interaction**						
Mutual grooming	1.44 ± 2.18	1.75 ± 2.44	1.23 ± 2.03	2.30 ± 2.83	0.79 ± 1.66	0.55 ± 1.70
Playing	0.23 ± 0.77	0.41 ± 1.20	0.16 ± 0.67	0.90 ± 1.84	0.15 ± 0.59	0.02 ± 0.21
Pen interaction	0.89 ± 1.79	0.78 ± 1.62	0.42 ± 1.22	1.42 ± 2.18	0.18 ± 0.69	0.02 ± 0.23
Sexual behavior	0.02 ± 0.19	0.02 ± 0.21	0.01 ± 0.20	0.08 ± 0.43	0.01 ± 0.18	0.00 ± 0.07
**Negative interaction**						
Cross-sucking	1.28 ± 2.13	0.39 ± 1.01	0.32 ± 0.96	1.78 ± 2.62	0.18 ± 0.64	0.06 ± 0.38
Stereotypies	0.21 ± 0.90	0.24 ± 0.97	0.31 ± 0.97	0.41 ± 1.53	0.17 ± 0.67	0.15 ± 0.76
Head butting	0.54 ± 1.17	0.93 ± 1.62	0.28 ± 0.84	1.60 ± 2.15	0.34 ± 0.97	0.04 ± 0.28
Self-grooming	2.32 ± 2.57	2.96 ± 2.59	3.26 ± 3.35	3.59 ± 3.30	2.43 ± 3.02	1.94 ± 2.50
Non-visible	4.32 ± 18.63	4.22 ± 19.34	3.81 ± 18.29	3.97 ± 18.20	5.45 ± 20.98	3.55 ± 17.99

EM, early morning; LM, late morning; EA, early afternoon; LA, late afternoon; EV, evening; NI, night.

^a^Time slots.

The mixed-model analysis (Table 4) shows the incidence of the main effects (diet, sex, and timing) and their interactions for each target behavior (Table 5). Diet-influenced (*P* < 0.05) behaviors include eating liquid, running, cross-sucking, licking, olfactory investigation, and mutual grooming. Sex influenced (*P* < 0.05) eating liquid, sternal recumbency, lateral recumbency, sternal ruminating, moving, running, licking, and sexual behavior. Timing influenced (*P* < 0.05) all behaviors.

**Table 4:** ANOVA reporting the *F*-statistics and *P*-values of all the main effects and interactions of all behaviors expressed as a percentage of recordings.

**Behavior**	**Day**		**Diet (D)**		**Sex (S)**		**Timing (T)**		**D** ^*^S		**D** ^*^T		**S** ^*^T		**D** ^*^S ^*^T	
	***F***	***P*** **-value**	***F***	***P*** **-value**	***F***	***P*** **-value**	***F***	***P*** **-value**	***F***	***P*** **-value**	***F***	***P*** **-value**	***F***	***P*** **-value**	***F***	***P*** **-value**
**Ingestion**																
Eating solid	11.85	<0.001	2.54	0.091	0.87	0.352	518.56	<0.001	4.32	0.013	5.14	<0.001	1.73	0.124	1.87	0.044
Eating liquid	15.35	<0.001	12.15	0.000	34.70	<0.001	1,441.77	<0.001	8.81	<0.001	11.52	<0.001	33.18	<0.001	6.37	<0.001
Drinking	34.45	<0.001	1.82	0.174	0.18	0.675	12.51	<0.001	1.76	0.171	1.38	0.184	1.95	0.083	1.27	0.240
**Resting**																
Sternal recumbency	52.82	<0.001	0.37	0.690	4.76	0.029	400.54	<0.001	1.91	0.148	1.14	0.329	4.55	<0.001	0.89	0.543
Lateral recumbency	86.53	<0.001	2.38	0.105	9.67	0.002	94.87	<0.001	0.31	0.734	2.68	0.003	2.37	0.037	0.81	0.616
Rest standing	60.85	<0.001	1.32	0.279	0.16	0.692	614.74	<0.001	0.10	0.904	4.19	<0.001	0.94	0.456	1.57	0.108
**Ruminating**																
Standing ruminating	8.35	<0.001	1.02	0.371	0.16	0.691	4.20	0.001	1.70	0.182	1.65	0.087	2.21	0.051	1.47	0.144
Lateral ruminating	31.69	<0.001	1.04	0.362	1.45	0.229	87.23	<0.001	1.02	0.360	1.88	0.044	0.70	0.620	2.63	0.003
Sternal ruminating	45.27	<0.01	0.41	0.668	4.52	0.034	350.33	<0.001	0.70	0.498	4.88	<0.001	9.57	<0.001	1.58	0.105
**Movement**																
General agitation	6.51	<0.001	2.39	0.105	2.06	0.152	61.05	<0.001	0.47	0.626	1.59	0.103	2.41	0.034	5.13	<0.001
Standing/lying	2.68	<0.001	0.15	0.862	2.76	0.097	3.50	0.004	0.27	0.761	1.14	0.325	1.12	0.345	1.78	0.059
Moving	27.49	<0.001	0.12	0.887	4.50	0.034	98.69	<0.001	3.65	0.026	1.81	0.054	4.77	<0.001	2.48	0.006
Running	3.79	<0.001	5.05	0.011	17.61	<0.001	59.83	<0.001	4.13	0.016	3.51	<0.001	10.39	<0.001	4.24	<0.001
**Environmental interaction**																
Olfactory investigation	91.16	<0.001	3.82	0.030	0.47	0.492	228.15	<0.001	1.38	0.251	2.37	0.009	0.76	0.579	1.77	0.060
Object playing	1.66	0.043	1.59	0.217	0.04	0.836	9.07	<0.001	5.05	0.006	1.03	0.416	0.97	0.432	1.46	0.148
Licking	20.57	<0.001	3.71	0.033	4.95	0.026	451.73	<0.001	1.81	0.164	5.24	<0.001	6.61	<0.001	3.23	<0.001
**Positive interaction**																
Mutual grooming	10.82	<0.001	3.42	0.042	0.48	0.487	54.51	<0.001	0.77	0.464	0.25	0.991	1.30	0.261	1.32	0.214
Playing	31.09	<0.001	0.80	0.456	1.32	0.251	57.59	<0.001	0.66	0.518	1.68	0.080	0.51	0.769	0.57	0.843
Pen interaction	32.87	<0.001	0.75	0.477	1.96	0.161	99.07	<0.001	10.30	<0.001	1.56	0.111	1.62	0.151	4.45	<0.001
Sexual behavior	2.38	0.001	1.14	0.329	5.69	0.017	3.55	0.003	2.80	0.061	1.03	0.412	4.59	<0.001	0.90	0.531
**Negative interaction**																
Cross-sucking	18.21	<0.001	5.51	0.008	3.15	0.076	166.64	<0.001	3.91	0.020	8.54	<0.001	5.77	<0.001	8.31	<0.00
Stereotypies	5.62	<0.001	0.77	0.471	0.00	0.961	5.83	<0.001	0.06	0.939	1.11	0.349	0.34	0.888	0.82	0.605
Head butting	23.06	<0.001	0.34	0.712	1.38	0.240	118.59	<0.001	0.59	0.554	1.22	0.271	1.07	0.375	0.88	0.556
Self-grooming	15.86	<0.001	0.34	0.711	13.80	<0.001	34.63	<0.001	1.66	0.191	1.18	0.298	1.50	0.187	1.56	0.113

**Table 5:** Least square means of behaviors, expressed as a percentage of recordings for the main effects considered in the ANOVA.

**Behavior**	**Diet**				**Sex**			**Timing** ^*****A*****^						
	**CTR**	**T3**	**T6**	**SE**	***F***	**M**	**SE**	**EM**	**LM**	**EA**	**LA**	**EV**	**NI**	**SE**
**Ingestion**														
Eating solid	5.016	4.211	4.655	0.247	4.760	4.494	0.194	6.412^c^	8.343^b^	1.754^d^	9.989^a^	0.855^e^	0.410^e^	0.219
Eating liquid	0.701^b^	0.844^a^	0.648^b^	0.028	0.827^a^	0.635^b^	0.022	2.125^b^	0.000^c^	0.000^c^	2.260^a^	0.000^c^	0.000^c^	0.032
Drinking	0.253	0.125	0.172	0.048	0.195	0.172	0.037	0.135^b^	0.289^b^	0.159^a^	0.318^a^	0.140^b^	0.059^b^	0.038
**Resting**														
Sternal recumbency	38.81	38.22	37.24	1.344	39.79^a^	36.40^b^	1.055	29.50^d^	40.49^c^	40.74^c^	17.34^e^	54.64^a^	45.86^b^	0.979
Lateral recumbency	10.00	7.945	7.130	0.994	6.573^b^	10.15^a^	0.780	5.893^c^	6.242^c^	11.58^b^	1.92^d^	10.89^b^	13.63^a^	0.707
Rest standing	10.81	10.190	8.797	0.931	9.719	10.14	0.730	14.33^b^	10.69^c^	5.371^d^	21.36^a^	5.752^d^	2.09^e^	0.598
**Ruminating**														
Standing ruminating	0.070	0.113	0.076	0.022	0.081	0.091	0.017	0.137^a^	0.074^a^	0.059^a^	0.149^a^	0.032^b^	0.064^a^	0.024
Lateral ruminating	2.687	2.388	1.848	0.432	2.007	2.608	0.339	2.508^b^	1.048^c^	2.911^b^	0.481^d^	1.397^c^	5.501^a^	0.306
Sternal ruminating	12.72	13.97	13.35	0.963	14.53^a^	12.16^b^	0.756	13.60^c^	7.583^e^	18.21^b^	6.129^e^	9.870^d^	24.69^a^	0.655
**Movement**														
General agitation	0.108	0.124	0.083	0.013	0.094	0.116	0.010	0.158^c^	0.064^b^	0.006^c^	0.379^a^	0.021^c^	0.000^b^	0.019
Standing/lying	0.223	0.236	0.216	0.025	0.249	0.201	0.019	0.230^a^	0.228^a^	0.223^a^	0.158^b^	0.312^a^	0.199^b^	0.028
Moving	0.666	0.715	0.684	0.068	0.772^a^	0.604^b^	0.054	0.723^b^	0.826^b^	0.494^c^	1.580^a^	0.395^c^	0.111^d^	0.061
Running	0.071^b^	0.126^a^	0.091^a^	0.012	0.125^a^	0.066^b^	0.010	0.018^c^	0.093^b^	0.026^b^	0.357^a^	0.082^b^	0.000^c^	0.017
**Environmental interaction**														
Olfactory investigation	5.716^a^	5.271^a^	4.510^b^	0.324	5.037	5.294	0.254	5.686^b^	6.428^b^	4.408^c^	9.700^a^	3.376^d^	1.395^e^	0.254
Object playing	0.047	0.100	0.099	0.025	0.085	0.079	0.019	0.154^a^	0.085^a^	0.066^b^	0.159^a^	0.026^b^	0.000^b^	0.024
Licking	4.913^b^	6.753^a^	6.195^a^	0.489	6.583^a^	5.325^b^	0.384	8.161^b^	6.909^c^	5.131^d^	12.23^a^	2.611^e^	0.678^f^	0.334
**Positive interaction**														
Mutual grooming	1.644	1.234	1.030	0.175	1.232	1.373	0.138	1.315^b^	1.661^b^	1.210^c^	2.294^a^	0.797^d^	0.540^d^	0.127
Playing	0.284	0.338	0.289	0.032	0.282	0.325	0.025	0.230^c^	0.407^b^	0.161^c^	0.862^a^	0.141^c^	0.021^d^	0.041
Pen interaction	0.576	0.711	0.682	0.082	0.723	0.590	0.065	0.905^b^	0.862^b^	0.450^c^	1.508^a^	0.188^d^	0.027^d^	0.069
Sexual behavior	0.029	0.010	0.014	0.009	0.005^b^	0.031^a^	0.007	0.018^a^	0.014^b^	0.010^b^	0.053^a^	0.010^b^	0.002^b^	0.010
**Negative interaction**														
Cross-sucking	0.927^a^	0.483^ab^	0.716^a^	0.093	0.804	0.614	0.073	1.390^b^	0.383^c^	0.326^c^	1.902^a^	0.204^c^	0.047^d^	0.076
Stereotypies	0.169	0.172	0.394	0.150	0.241	0.249	0.118	0.199^b^	0.249^a^	0.304^a^	0.384^a^	0.177^b^	0.156^b^	0.093
Head butting	0.592	0.641	0.568	0.062	0.558	0.642	0.049	0.539^c^	0.852^b^	0.256^d^	1.595^a^	0.322^d^	0.040^e^	0.059
Self-grooming	2.854	3.068	3.077	0.220	3.472	2.527	0.173	2.490^c^	3.320^b^	3.562^a^	3.837^a^	2.664^c^	2.125^d^	0.164

CTR, control; T3, 3% of hempseed cake inclusion; T6, 6% of hempseed cake inclusion; F, female; M, male; EM, early morning; LM, late morning; EA, early afternoon; LA, late afternoon; EV, evening; NI, night; SE, standard error.

^*A*^Time slots.

^a−*f*^Values within a row with different superscripts differ significantly at a P-value of <0.05.

**Figure 2:**
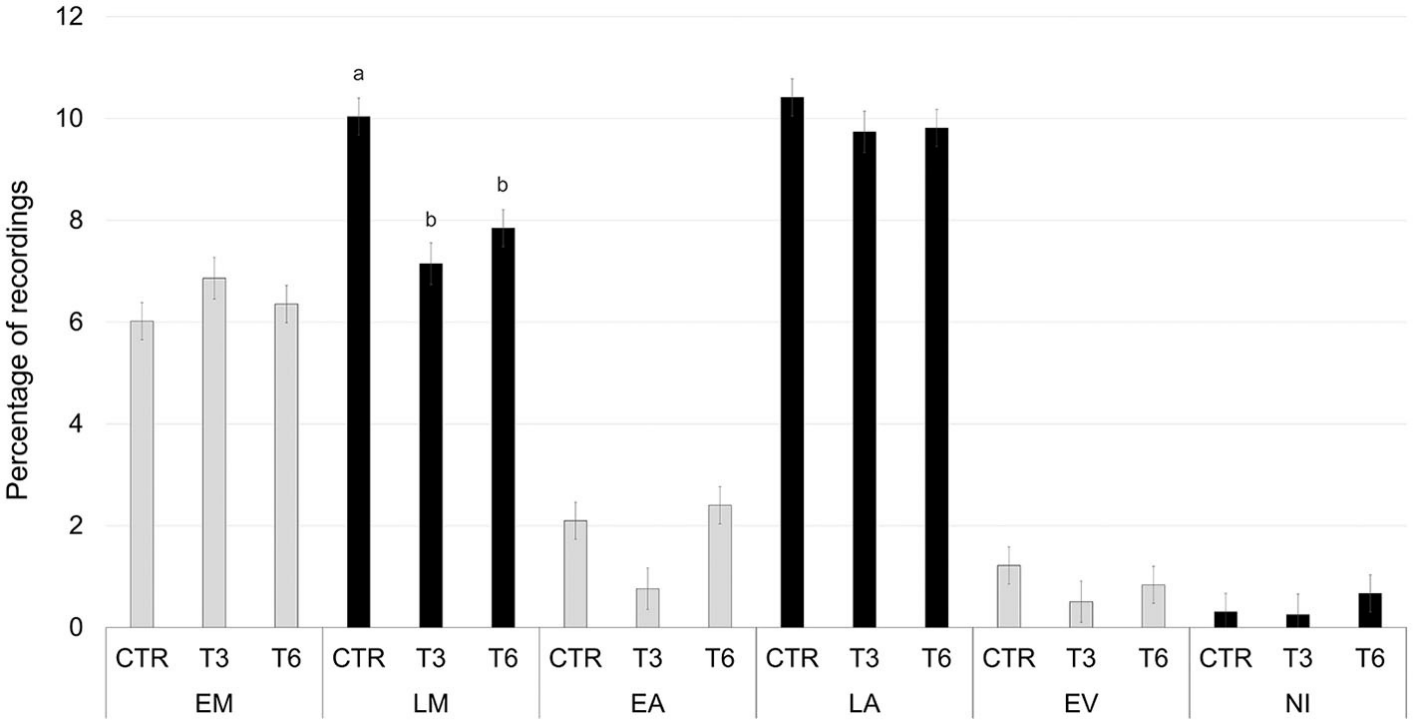
Least square means of the interaction of diet (CTR, 0% of HSC; T3, 3% of HSC; T6, 6% of HSC), and timing (EM, early morning; LM, late morning; EA, early afternoon; LA, late afternoon; EV, evening; NI, night) on licking behavior expressed as a percentage of recordings. Black lines represent SE. Different letters differ statistically (*P* < 0.05).

**Figure 3:**
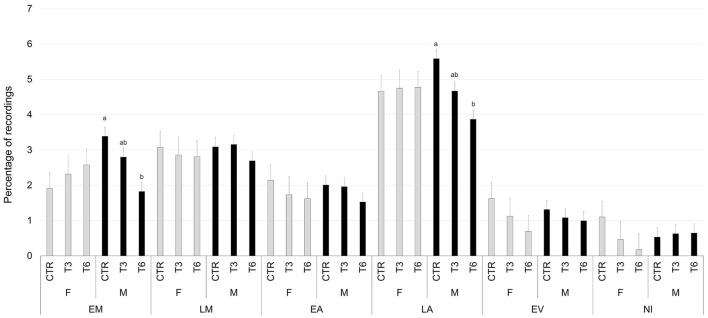
Least square means of the interaction of diet (CTR, 0% of HSC; T3, 3% of HSC; T6, 6% of HSC), sex (F, female; M, male), and timing (EM, early morning; LM, late morning; EA, early afternoon; LA, late afternoon; EV, evening; NI, night) on cross-sucking behavior expressed as a percentage of recordings. Black lines represent SE. Different letters differ statistically (*P* < 0.05).

**Figure 4:**
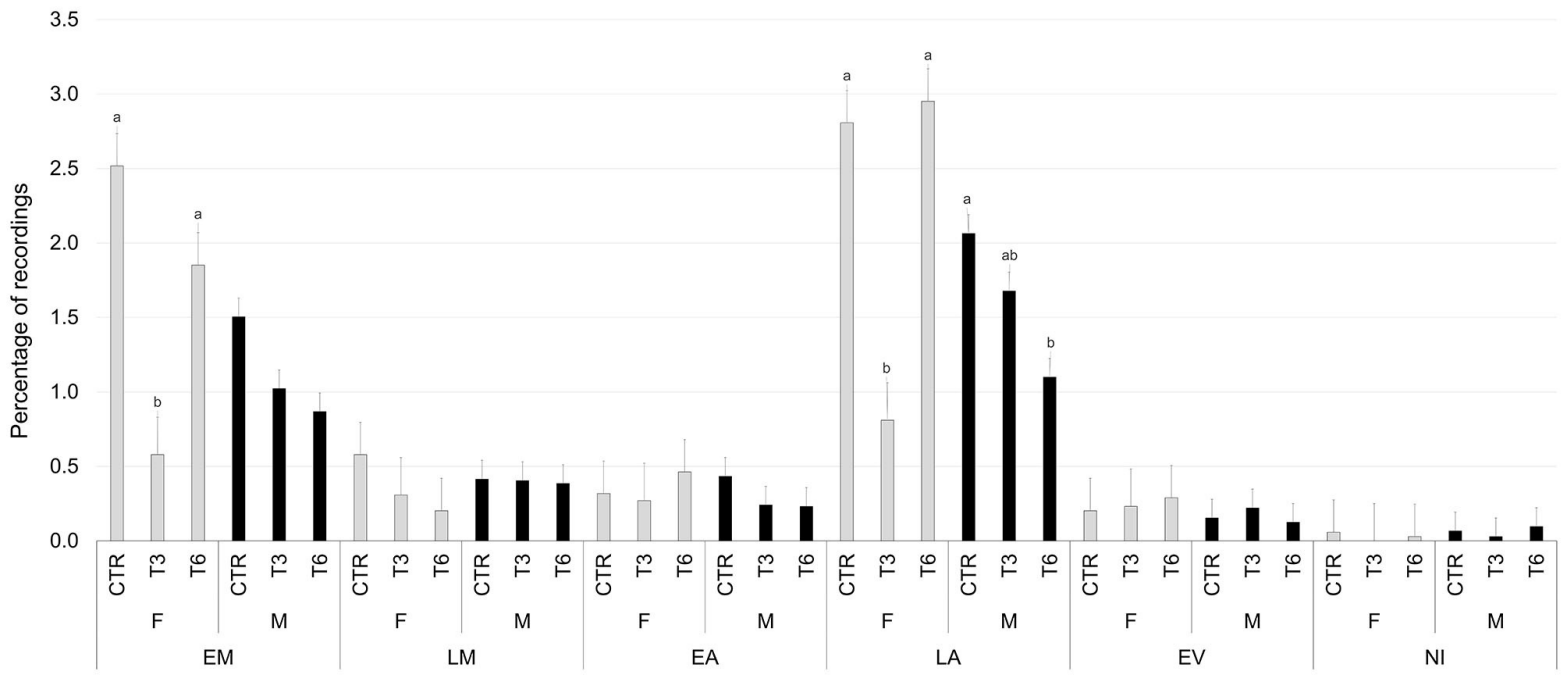
Least square means of the interaction of diet (CTR, 0% of HSC; T3, 3% of HSC; T6, 6% of HSC), and timing (EM, early morning; LM, late morning; EA, early afternoon; LA, late afternoon; EV, evening; NI, night) on eating solid behavior expressed as a percentage of recordings. Black lines represent SE. Different letters differ statistically (*P* < 0.05).

**Figure 5:**
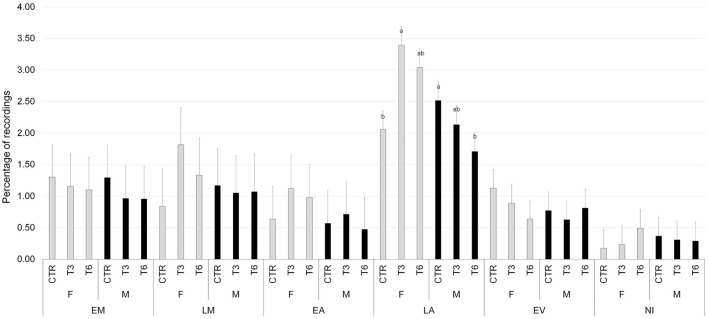
Least square means of the interaction of diet (CTR, 0% of HSC; T3, 3% of HSC; T6, 6% of HSC), sex (F, female; M, male), and timing (EM, early morning; LM, late morning; EA, early afternoon; LA, late afternoon; EV, evening; NI, night) on positive interaction behavior expressed as a percentage of recordings. Black lines represent SE. Different letters differ statistically (*P* < 0.05).

**Figure 6:**
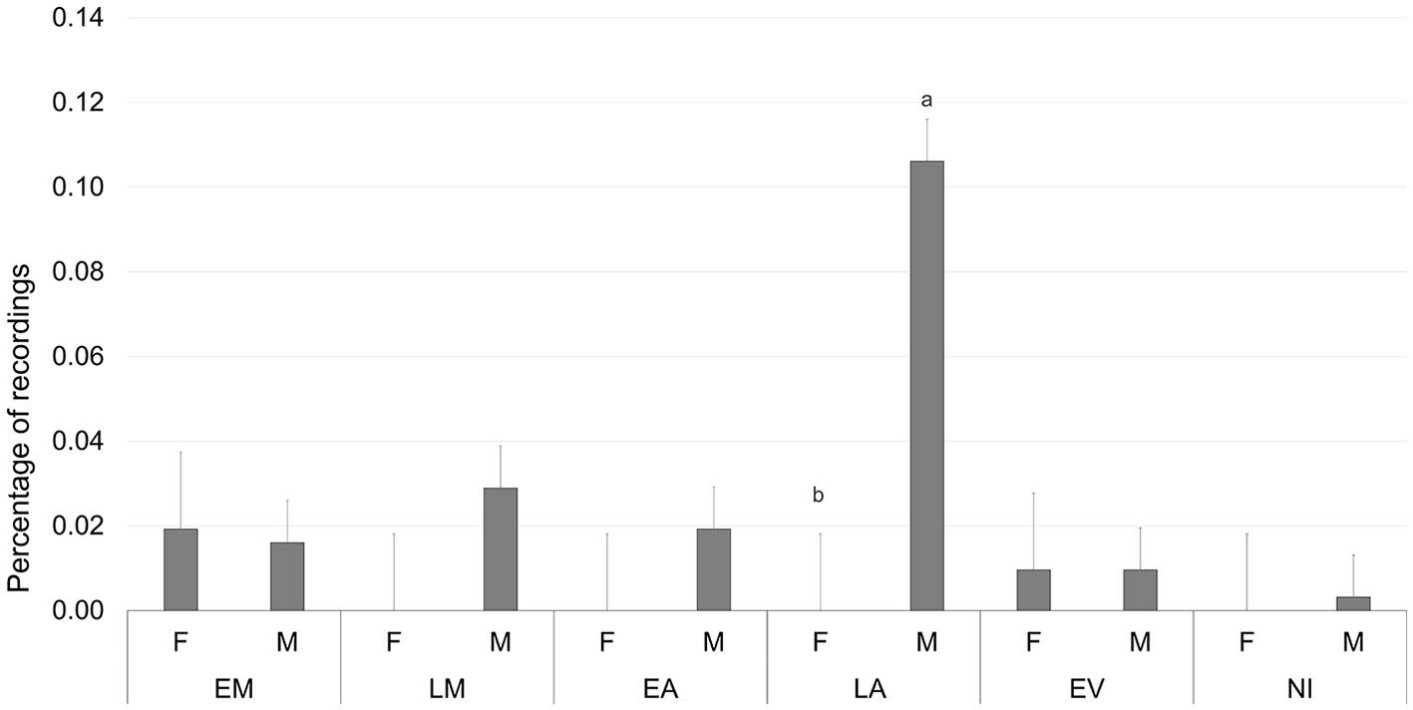
Least square means of the interaction of diet (CTR, 0% of HSC; T3, 3% of HSC; T6, 6% of HSC), sex (F, female; M, male), and timing (EM, early morning; LM, late morning; EA, early afternoon; LA, late afternoon; EV, evening; NI, night) on movement behavior expressed as a percentage of recordings. Black lines represent SE. Different letters differ statistically (*P* < 0.05).

**Figure 7:**
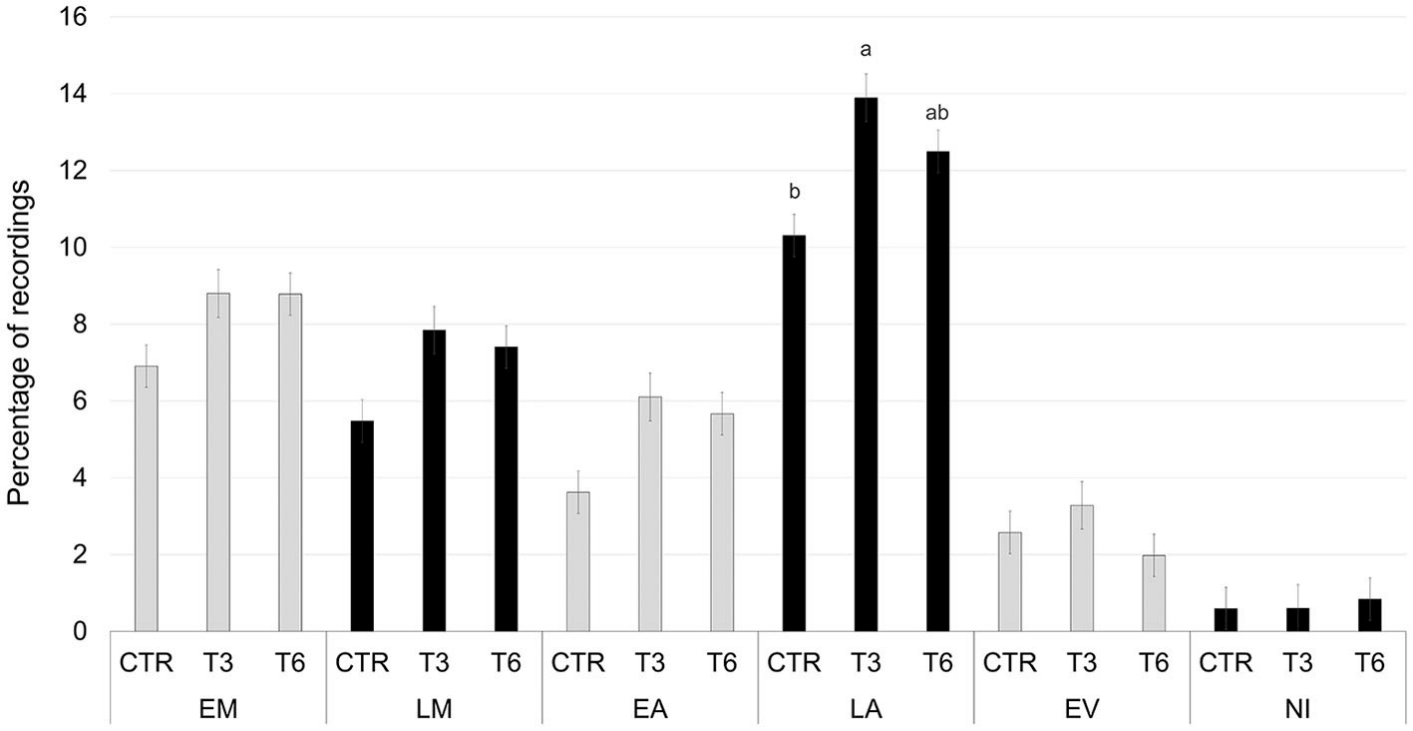
Least square means of the interaction of sex (F, female; M, male), and timing (EM, early morning; LM, late morning; EA, early afternoon; LA, late afternoon; EV, evening; NI, night) on sexual behavior expressed as a percentage of recordings. Black lines represent SE. Different letters differ statistically (*P* < 0.05).

Figures 2–6 report the most relevant interactions among the main effects that were significant (*P* < 0.05). The inclusion of 3% and 6% of HSC significantly increased the time spent running and licking (Figure 2) and decreased the amount of time carrying out olfactory investigation and cross-sucking (Figure 3). Both sexes expressed more time cross-sucking during the early morning and late afternoon, but mostly in the latter after the second milk administration. Male calves reduced the time spent on cross-sucking as the HSC inclusion increased in the diet. For female calves, this reduction only happened for the T3 group. Meanwhile, the T6 and CTR groups were not influenced by the diet (Figure 3). Females spent significantly greater time than males eating liquid, resting, ruminating in sternal recumbency, moving, running, and licking, whereas males spent longer duration in lateral recumbency and sexual behavior. The CTR group spent more time eating solids in the late morning than T3 and T6 (Figure 4). Calves that received the diet with the HSC inclusion finished all the solid feed right after the first meal, whereas the CTR group always had some leftovers in the feeder, and they tended to eat more slowly. The diet did not influence the positive interaction behavior, but sex and timing did (Figure 5). This behavior was observed, in particular, during the late afternoon. For this time period, male calves decreased the time of positive interaction inside the pen when the HSC was included in the diet, whereas the same effect was not noticed in the female calves, which spent the same amount of time interacting positively regardless of the diet. During this trial, both male and female calves showed a different time budget for movement behavior (Figure 6). Even though both female and male calves were more active during the late afternoon, the HSC inclusion had opposite effects on the different sexes. Female calves of T3 and T6 spent more time (*P* < 0.05) on movement than those in the CTR group. On the contrary, male calves decreased (*P* < 0.05) their time spent on movement when the HSC inclusion increased in the diet. Although sexual behavior was the least noticed of the positive interaction behaviors, there was a statistical difference between sexes (Figure 7), showing that male calves expressed more sexual behavior than females, mostly in the late afternoon.

## 4 Discussion

As hemp has been a controversial ingredient due to its THC and CBD content, its use as an ingredient in calves' diets should not change the behavior the animals normally show when they are fed with conventional diets. Resting is the most frequently seen behavior in veal calves in the literature (11), and this study supports this fact. The amount of time spent on resting depends, above all, on the space allowance (12). According to EU regulations, housed calves should have the possibility of lying simultaneously with a minimum space allowance of 1.5 m^2^ for each calf of a live weight of <150 kg (Council Directive 98/58/EC). In this trial, the space for each animal was 1.9 m^2^, so this behavior took place under normal standards. Lateral recumbency is considered an abnormal posture when it lasts for long periods (13). Positively, in this trial, lateral recumbency was seen less than sternal recumbency. Furthermore, it was observed that recumbency increased steadily as day turned into night. This could be explained by the literature since this inactive behavior was found to be more common at night than during the day (11). Rumination was the second most seen behavior in veal calves, and it is known that it allows the use of solid feed and may be affected by the type of feed available (14). Not considering the early morning and late afternoon, the amount of time spent doing cross-sucking in the other time slots was less than the normal percentage of 0.5 (15). This may be due to the fact that group housing normally increases this behavioral disturbance, even though this type of housing system is beneficial for the calves' welfare (16). However, the fact that this behavior was noticed most after the evening meal is normal because cross-sucking occurs strongly within 10–15 min after milk feeding (16). Stereotypical behaviors in cattle have been generally highlighted more in traditional tie-stalls than in loose-housing systems, and their expression also seems to increase when restricted feed is provided (17). The overall small amount of stereotypies observed in this study is likely to depend on the situation of loose housing despite the restricted feeding typical of rearing systems for fattening calves.

Regarding the more time spent eating solids in the late morning by the CTR group and knowing that the solid feed was given right after the liquid feed, it may be that the HSC inclusion in the milk somehow increased the appetite of the calves; thus, they ate the solid feed faster than the CTR group. Considering this hypothesis, HSC may also increase feed intake when offered *ad libitum*, but the only study to date that used 3% of HSC in the concentrate of Holstein veal calves did not find any increase in feed intake (18). HSC inclusion in females' diets made them more active, whereas males did not follow the same pattern, maybe due to a hormone interaction. The effect of the diet diminishing the duration of males doing cross-sucking is positive since it is a non-nutritive behavior that normally disappears when the calves are weaned (16). Even though sexual behavior is one of the least seen behaviors, it is important to discuss it because it is a big part of the calves' life that develops around the age range of 4–6 months (19). It was normal that the calves in this experiment expressed this behavior because they reached 6 months of age at the end of the trial. In addition, these results agree with the study of van Ek (19), who reported 0.18% of time spent in this behavior by calves of 4–6 months of age and also found that bull calves had more sexual activity than females and that they displayed more sexual interaction during the morning (07.00 a.m.−11.00 a.m.) and the afternoon (4.00 p.m.−7.00 p.m.) (19).

In conclusion, this study demonstrated that hemp seed cake had little effect on calves' behavior and that calves, in general, spend most of their time resting and ruminating, as they normally do with conventional diets. HSC inclusion increased the appetite for solid food and licking behavior during the late afternoon. The highest hempseed inclusion increased the female calves' movement in the late afternoon. Male calves decreased their positive interaction, movement, and cross-sucking in the late afternoon as the inclusion of HSC increased. Considering the findings given above, the inclusion of hempseed cake into veal calves' diet can be suggested, but further studies on different breeds and individual ages and the relationship between the cannabinoid content of hemp would be interesting for a better understanding of this novel ingredient.

## Data availability statement

The original contributions presented in the study are included in the article/supplementary material, further inquiries can be directed to the corresponding author.

## Ethics statement

The animal study was approved by Ethical Committee at the University of Padova. The study was conducted in accordance with the local legislation and institutional requirements.

## Author contributions

SA: Formal analysis, Investigation, Writing – original draft, Writing – review & editing. NG: Investigation, Writing – review & editing. VT: Visualization, Writing – review & editing. ER: Data curation, Methodology, Validation, Writing – review & editing. CS: Data curation, Formal analysis, Writing – review & editing. LB: Conceptualization, Resources, Supervision, Validation, Visualization, Writing – review & editing.
